# Assessments of sarcopenia and its associated factors in community-dwelling middle-aged and older Chinese adults in Kelantan, Malaysia

**DOI:** 10.1038/s41598-023-34668-w

**Published:** 2023-05-09

**Authors:** Leng Huat Foo, Yin Siew Wen, Azidah Abdul Kadir

**Affiliations:** 1 School of Health Sciences, Universiti Sains Malaysia, Health Campus, Kubang Kerian, Kelantan, Malaysia; 2 School of Medical Sciences, Universiti Sains Malaysia, Health Campus, Kubang Kerian, Kelantan, Malaysia

**Keywords:** Medical research, Risk factors

## Abstract

Sarcopenia is an emerging public health problem worldwide, but very limited information exits concerning the influence of lifestyle factors and inflammation on sarcopenia among community-dwelling older populations in Asia, including Malaysia. A total of 230 apparently healthy community-dwelling middle-aged and older Chinese adults were included in the study. Validated questionnaires were used to assess dietary and lifestyle practices, while pro-inflammatory cytokine status was assessed by blood interleukin-6 concentrations (IL-6). Sarcopenia risk was assessed by the newly revised diagnostic criteria of the Asian Working Group for Sarcopenia. The prevalence of sarcopenia was 12.5% with similar proportions of males and females. Multivariate logistic regression analysis showed that older age and higher concentrations of pro-inflammatory cytokine levels of IL-6 were significantly associated with a greater risk of sarcopenia, after adjustments for potential known biological and body composition factors. The present findings indicate that older adults aged 70 years and above with higher inflammation levels had a significantly increased risk of sarcopenia. Hence, effective dietary and lifestyle intervention strategies should emphasize reducing the inflammation associated with aging to prevent the rapid loss of muscle mass and strength that can lead to sarcopenia.

## Introduction

Sarcopenia is a disease defined by the age-related progressive decline of skeletal muscle mass, characterised by accelerated loss of strength and reduced physical performance^1,2^. Sarcopenia significantly increases the risk of fragility fracture, disability, and functional impairment that all require a longer hospital admission and/or long-term care placement. Poor health-related quality of life, and mortality risk, among the older populations^2–4^, imposes a higher burden on patients and healthcare systems.

Although the reported prevalence of sarcopenia varies greatly due to differences in the methods of assessment, diagnostic classifications and cut-off points used, the estimated overall prevalence ranges between 0.2% and 86.5% based on a recent systematic review of published studies conducted worldwide^5^. A similarly high prevalence of sarcopenia has been reported among older populations in the Asian region^1,6^. For instance, in a recent population study in China using a BIA-derived muscle mass measurement to define sarcopenia, the prevalence of the condition among multi-ethnic middle-aged adults and elderly was 19.3%^6^. An observation was likewise reported among healthy older adults of multiethnic groups in Malaysia, where approximately 3 out of every 5 older adults aged 60 years and above had sarcopenia^7^. Of note, a high prevalence of sarcopenia (47%) was found among community-dwelling older adults aged 60 years and above living in long-term care facilities in the urban region of Klang Valley in Malaysia^8^. Hence, sarcopenia is regarded as an emerging public health challenge in Asia because it is rising rapidly as life expectancy increases among Asian populations^1^.

Although the risk of sarcopenia generally increases with age^5^, the pathophysiology is not well elucidated and is likely multifactorial. The risk of sarcopenia entails multiple body systems—muscle, neural and hormonal showing changes associated with the aging process^9^. Significant progress has been made over the past few years in understanding the mechanisms associated with the risk of development of sarcopenia. Extensive studies have documented that the risk has a significant association with the progression of muscle atrophy among older adults^2^. Several studies have shown that the quality of muscle fibres gradually deteriorates and the peak power, speed and elasticity of muscles declines with aging^10^. A decline in the quality and functionality of muscle fibres could possibly be explained by several physiological changes associated with ageing such as the loss of anabolic stimuli caused by a decline in testosterone, and other anabolic hormones^2^. In addition, it has been proposed that age-associated subclinical inflammation could underlie the pathophysiology of sarcopenia risk^11,12^, with higher levels of inflammatory cytokines significantly associated with poor muscle strength and mass^12^. For example, high levels of pro-inflammatory cytokines are indirectly responsible for the generation of reactive oxygen species in skeletal muscle cells, which occurs through the stimulation of pro-oxidant expression via a NF-κB signalling pathway^13^. Interleukin-6 (IL-6) is a major proinflammatory cytokine produced in a wide variety of tissues, including activated leukocytes, adipocytes, and endothelial cells. IL‐6 is regarded as an upstream inflammatory cytokine exert a range of pleiotropic effects on inflammation, immune response, and hematopoiesis, bone metabolism and embryonic development^14^. Increased levels of IL-6 have been associated with muscle loss, muscle strength and sarcopenia risk^15,16^. Hence, IL-6 has been implicated in the generation and propagation of chronic inflammation and it has been increasingly used for the evaluation of inflammatory severity in clinical laboratory examinations. Although some have indicated that oxidative stress and molecular inflammation could contribute to the loss of muscle mass and strength^11,12^, findings are inconclusive examining the relationship between oxidative stress, inflammation and sarcopenia risk among apparently healthy community-dwelling older populations in a population-based epidemiological setting.

It is generally agreed that nutrition and lifestyle factors such as dietary nutrients intake, physical activity, sedentary practices, and smoking play crucial role in maintaining skeletal muscle mass in the older population^17^, but there is little data concerning the influence of these lifestyle factors on sarcopenia risk among community-dwelling middle-aged and older populations in Asia^18–21^. Most studies have been on older Caucasian populations, whose dietary and lifestyle practices are different than older populations in Asia. Likewise, no studies have been conducted on older adults in Malaysia or elsewhere in Asia, designed to investigate a comprehensive range of modifiable diet and lifestyle factors that may affect the loss of skeletal muscle mass and strength associated with sarcopenia risk such as body composition, dietary intake, and lifestyle choices. Understanding these factors and the interactions associated with the risk of skeletal muscle mass and function among community-dwelling older populations is urgently needed, as the prevalence of sarcopenia is increasingly recognised as a public health challenge among older populations worldwide, including Malaysia. It predicts serious implications for the overall health of individuals, and national economic and social development. Furthermore, an assessment of the blood biochemical levels of inflammatory markers for sarcopenia risk would enable the identification of the associated factors. Subsequently, it could be used for effective nutrition and lifestyle interventions of the dietary and lifestyle modifiable factors associated with the lower risk of age-related subclinical inflammation associated with sarcopenia. Hence, maintaining and preventing excessive age-related muscle mass and function loss among older populations should be accorded high priority in public health policy. Moreover, no studies have been carried-out on community-dwelling apparently healthy middle-aged adults and the elderly of Asian origin, with the variables that affect skeletal muscle mass associated with sarcopenia risk such as body composition, dietary intake, lifestyle physical activity, and blood inflammation markers. The primary aim of the present study was to investigate the relationship between dietary nutrient intake and lifestyle practices on sarcopenia risk in apparently healthy community-dwelling middle-aged and older Chinese adults aged 50 years and above in Kelantan, Malaysia. The secondary aim was to elucidate the possible mechanistic pathway between blood inflammatory interleukin-6 marker and sarcopenia risk, after taking into account biological, dietary and lifestyle factors.

## Materials and methods

### Study design

The present population-based study was designed to investigate the influence of dietary and lifestyle practices on metabolic syndrome risk in middle-aged and older adults of Chinese-origin aged 50 years and above in the urban communities in Kelantan. A convenience sampling method was used to recruit all potential participants in the study. Several approaches were used for the invitation of the study participants namely, advertisements, community announcements, and peer-to-peer referrals in the community areas. Prior to the study, the nature, purpose, possible risks, procedures and requirements were explained carefully to participants. Recruitment was based on very strict inclusion and exclusion criteria, according to which all participants had to be apparently healthy community dwelling person free of any disabilities that would interfere with their daily living activities or cognitive impairment. They had to be capable of walking one block or climbing a flight of stairs without rest while free from any known chronic disease conditions (a recent episode of acute illness, cardiovascular, diabetes, kidney, liver diseases, rheumatoid arthritis), major depression or alcoholism^22,23^ during the study period. Moreover, participants were excluded from the present study if they had certain conditions or any clinical signs of disease that could impair being physically active and/or currently or were taking any medications known to affect glucose, and lipid and bone metabolism. At the end of the study, a total of 232 apparently healthy middle-aged and older adults successfully completed the study. However, for the blood sample analysis of the blood interleukin-6 (IL-6) marker, due to the extremely low levels of serum IL-6 levels of two females, only a total of 230 participants, comprising 70 males and 160 females were included.

### General characteristics, health status and socio-demographic assessments

All participants were required to complete a validated structured questionnaire of the general characteristic, including health and socio-demographic status assessments, with the assistance of a graduate research assistant. In addition, menstrual status was also recorded for females.

### Assessments of body composition and muscular strength of the handgrip

Anthropometric measurements such as body weight, height, hip and waist circumference were performed using the standardized procedures recommended by the World Health Organization^24^. All measurements were taken twice, however if they differed by more than 1.0 cm or 1.0 kg, a third measurement was taken and the mean of the two closest measurements was recorded. Handgrip strength was measured on both non-dominant and dominant hands using a hand-held dynamometer with adjustable widths (Lafayette Instrument company, model: J100105, Indiana USA). The width of the dynamometer was adjusted so the participant felt comfortable while squeezing the grip. During the assessment, participants were requested to be seated with 90° elbow flexion and were instructed to squeeze the dynamometer as hard as possible without pressing the instrument against the body or bending at the elbow. Two measurements were taken at 1- to 2-min intervals, with the higher handgrip strengths were used in the final analysis to determine sarcopenia risk based on diagnostic criteria.

Body composition profiles of the participants were assessed using a dual energy *X*-ray absorptiometry (DXA) device (Hologic, Waltham, MA, USA) at the Department of Medical Radiology, University Hospital, using software provided by the manufacturer. All bone measurements were performed by the same trained technician. The DXA was considered for the present study because this method of assessment distinguishes fat, bone mineral, and the lean tissues of the body. DXA is a widely used method of assessment for muscular measurements in sarcopenia research. For an assessment of the total body, each participant was required to wear light indoor clothing such as loose-fitting shorts and instructed to remove any metal and all clothes containing metal prior to the scan. The participant was placed in a supine position and asked to remain still during the bone scan. To minimize technical variations, the same technician analysed all bone data, using Hologic’s software. Quality control scans were performed each day. Prior to each scan, the densitometer was calibrated according to manufacturer' recommendations. During the course of the study, the precision of repeated measurements (CVs) using a manufacturer-supplied phantom, was < 1%, indicating satisfactory long-term stability of the instrument with no sign of drift. For ethical reasons, we did not perform repeat scans on the same participant after repositioning. To determine the sarcopenia risk, in the present study, two indicators of muscle mass were used namely, total lean body mass and appendicular muscle mass; both muscle masses were expressed in kilograms (kg).

### Blood biochemical marker of inflammation

About 5 mL of fasting venous blood was taken between 0800 and 1000 am during the DXA scan period. Blood samples of the participants throughout the study was collected by trained Medical Laboratory Technologists (MLTs) or staff nurses, under the supervision of medical personnel. The blood serum was separated and stored at − 80 °C immediately prior to analysis. The blood inflammatory marker of serum IL-6 was analysed using the ELISA assay kit (MyBioSource, San Diego, CA, USA) that employs a double antibody sandwich technique. All samples from the different participants were analysed at the same time in single batch assays in order to reduce inter-assay variation. Quality control assays assessing reproducibility identified the intra-assay CV and inter-assay CV as less than 15%.

### Dietary intake pattern and dietary behavioral practices and physical activity assessments

The dietary food intake of the participants was assessed by a validated a semi-quantitative food frequency questionnaire (FFQ) to reveal eating habits over the past one-year period. In addition, a comprehensive list of food photos was developed to help participants recall the food consumed while common household measures were used to help quantify food items, with the assistance of trained interviewers. Completed FFQs were then re-checked by a trained nutritionist for completeness and accuracy. A list of food commonly consumed by adults and the elderly in Kelantan was derived from dietary food intake lists taken from a pre-tested pilot study of 100 randomly adults and elderly in local areas of Kelantan. Furthermore, data on dietary behavior practices and dietary supplements were also gathered. As for lifestyle physical activity practices, a pre-piloted habitual physical activity (PA) over the previous 12 months was assessed, using a structured and detailed questionnaire for older persons. The questionnaire was developed by taking into consideration all physical activities (PA) commonly practised by adults in Malaysia as well as PA from different life domains such as leisure and household-based activities. Each participant was asked for detailed information on each activity such as how many hours per day for each one engaged. Total physical activity was expressed as total hours per day and the metabolic equivalents of each physical activity was gathered based on the revised Physical Activity Compendium Database published in 2011^25^. In addition, a screen-based sedentary lifestyle practice questionnaire was also gathered to assess screen-based behavioral practices such as time watching television, on the computer and smart phone.

### Assessments of sarcopenia risk

In the present study, sarcopenia risk assessment is carried-out based on the new revised diagnostic algorithm recommended by the Asian Working Group for Sarcopenia (AWGS)^1^. It is based on the presence of both low muscle mass and low muscle strength and/or low physical performance (Table 1).

**Table 1:** The new revised proposed definitions of the sarcopenia by the Asian Working Group for Sarcopenia (AWGS 2019). Source: Chen et al*.* (2020).

Diagnostic criteria of AWGS 2019	Measures	Cut-off points	
		Male	Female
Low muscle mass	ASM adjusted for height^2^	< 7.0 kg/m^2^	< 5.4 kg/m^2^
Low muscle strength	Handgrip strength	< 20th percentile of the study population	
Low physical performance	Gait speed	≤ 0.8 m/s	≤ 0.8 m/s

The algorithm for identifying and diagnosing older adults at-risk for sarcopenia is based on primary health care or community-based preventive services setting.

### Statistical analysis

Food nutrient intake data was analysed using the Diet and Nutrient Analysis Software Nutritionist Pro™, based on the Malaysia Food Composition Table^26^, apart from vitamin D values obtained from USDA food composition table^27^. Participants’ BMI was classified into normal weight, overweight or obese based on the WHO’s criteria^24^. High pro-inflammatory cytokine status was assessed by blood IL-6 concentrations above the gender-specific population median (IL-6 concentrations ≥ 1.10 pg/mL and ≥ 1.70 for males and female, respectively) and low pro-inflammatory cytokine status was defined as values lower than gender-specific median for males and females.

All variables were tested for normality against a standard normal distribution, using the Kolmogorov–Smirnov test and a test of homogeneity variance prior to any statistical comparisons. Since most dietary, lifestyle variables and blood IL-6 concentrations were not normally distributed; these variables were log transformed to derive a near-normal distribution and used in statistical analysis models. Descriptive statistics are presented as mean values ± SDs, and percentages and the number of participants, unless otherwise indicated. Sexes combined model was used to examine the relationships between cytokine IL-6 levels and body composition indicators of muscle mass and handgrip muscle strength. The results were used to assess sarcopenia risk as revealed in the continuous data and other explanatory continuous variables such as dietary and lifestyle. A multiple logistic regression model with stepwise elimination was then carried-out to assess the determinants of all factors investigated on sarcopenia risk, after adjusting for other potential confounding factors of biological, dietary and lifestyle factors. Where appropriate, the interaction between independent variables such as dietary nutrient intake, physical activity, body composition, menopausal status and socio-demographic status were also tested. Statistical analysis was conducted using the SPSS version 26.0 for Windows (SPSS Inc., Chicago, IL, US) and the statistical significance for all tests was defined by a *P*-value < 0.05.

### Ethical approval and informed to consent

The present study was approved by the Human Ethic Committee of the Universiti Sains Malaysia (USM) (Ref. No: USMKK/PPP/JEPeM [263.3(13)]. In addition, written informed consent was obtained from each participant prior to examination. All methods were performed following all relevant guidelines and regulations.

## Results

Table 2 shows the general characteristics of the study participants. A total of 230 participants, consisting of 70 males and 160 females were included in the analysis. The mean age was 58.2 ± 6.7 years, with male participants significantly older as compared to the females. The mean BMI of these participants was 23.5 ± 3.8 kg/m^2^, with a majority (63.5%) at the normal ranges of body weight. Almost one-third were at risk of overweight and obesity (30.4%), based on recommended BMI’s cut-off points used by WHO^21^. In terms of body composition indicators assessed by anthropometry and dual-energy χ-ray absorptiometry (DXA) measurements, males had significantly higher levels of all body composition indicators, except for adiposity indices. As expected, females tend to have significantly higher levels of total body fat (*P* < 0.01) and a percentage of body fat (*P* < 0.001) than the male participants. Males had a significantly higher level of handgrip muscle strength of both dominant and non-dominant hands (all *P* < 0.001) and total muscle mass, as expressed by lean body mass (*P* < 0.001), and height-adjusted appendicular muscle mass (*P* < 0.001) than females. In general, males were older, heavier and had higher muscle mass and handgrip muscle strength as compared to their female counterparts, while females had a higher level of adiposity. For dietary and lifestyle profiles, there was no significant different for most dietary nutrient intake between genders, except for total energy intake. Lifestyle practices showed a different pattern for males and females, whereby females spent a significantly higher total time in total physical activity (PA) than male participants (*P* < 0.001). There were no significant differences in only moderate-to-vigorous physical activity (MVPA) when, assessed either by total duration spent or metabolic equivalent levels based on the revised Compendium of Physical activity database^25^ as well as the total time spent in sedentary screen-based activities. Females had a significantly higher concentration of the pro-inflammatory cytokine marker of IL-6 levels than males (*p* < 0.001).

**Table 2:** General characteristics and body composition profile of the study population (N = 230).

	Males (n = 70)	Females (n = 160)	Total (n = 230)
	Mean ± SD		
*General characteristics*			
Age, *years*	59.7 ± 7.4*	57.5 ± 6.4	58.2 ± 6.7
Marital status			
Married^b^	65 (92.9)	124 (77.5)	189 (82.2)
Household size	3.4 ± 1.8	3.4 ± 1.7	3.4 ± 1.7
Number of child	2.7 ± 1.2	2.5 ± 1.4	2.6 ± 1.4
Age of menarche^a^, *years*	–	13.1 ± 1.6	–
Year of menopause^a^, *years*	–	45.7 ± 14.7	–
Year since menopause^a^, *years*	–	7.6 ± 6.5	–
*Body composition*			
Weight, *kg*	68.0 ± 10.6***	55.6 ± 9.8	59.4 ± 11.5
Height, *m*	1.7 ± 0.1***	1.6 ± 0.1	1.6 ± 0.1
Body mass index (BMI), *kg/m2*	24.6 ± 3.4**	23.0 ± 3.8	23.5 ± 3.8
Underweight^b^	2 (2.9)	12 (7.5)	14 (6.1)
Normal	42 (60.0)	104 (65.0)	146 (63.5)
Overweight	20 (28.6)	3.6 (22.5)	56 (24.3)
Obese	6 (8.6)	8 (5.0)	14 (6.1)
Waist circumference, *cm*	89.8 ± 8.1***	79.8 ± 9.1	82.8 ± 10.0
Hip circumference, *cm*	96.2 ± 6.3	95.1 ± 8.0	95.5 ± 7.5
Waist-to-hip ratio	0.9 ± 0.1***	0.8 ± 0.1	0.9 ± 0.1
Handgrip muscle strength, *kg*			
Dominant hand	40.8 ± 9.5***	25.5 ± 5.5	30.1 ± 9.9
Non-dominant hand	37.2 ± 8.9***	23.6 ± 4.9	27.7 ± 8.9
DXA-Lean body mass, *kg*	48.5 ± 6.1***	33.6 ± 4.3	38.1 ± 8.5
DXA-height-adjusted ASM, *kg/m*^2^	7.7 ± 0.8***	5.8 ± 0.8	6.4 ± 1.2
DXA-fat mass, *kg*	18.4 ± 5.5**	21.1 ± 6.0	20.3 ± 6.0
DXA-body fat percent, *%*	26.2 ± 4.9***	36.8 ± 5.1	33.6 ± 7.0
*Dietary and lifestyle profiles*			
Energy^c^, *Kcal/d*	2060.7** (1920.5; 2200.9)	1817.2 (1736.8; 1897.6)	1891.3 (1820.0; 1962.6)
Protein^c^, *g/d*	74.1 (67.5; 80.7)	67.3 (63.4; 71.2)	69.4 (66.0; 72.7)
Fat^c^, *g/d*	53.6 (48.3; 58.9)	52.1 (47.7; 56.6)	52.6 (49.1; 56.0)
Omega 3 FA^c^, *g*	1.7 (1.4; 2.0)	1.7 (1.4; 1.9)	1.7 (1.5; 1.9)
Omega 6 FA^c^, *g*	3.4 (2.9; 3.8)	3.3 (3.0; 3.6)	3.3 (3.0; 3.6)
Vitamin D^c^, *μg*	5.4** (4.6; 6.2)	4.1 (3.7; 4.5)	4.9 (4.1; 4.9)
Calcium^c^, *mg*	562.4 (500.2; 624.6)	531.5 (492.2; 570.8)	540.9 (507.9; 574.0)
Iron^c^, *mg*	20.2 (17.9; 22.5)	18.7 (17.4; 20.0)	19.1 (18.0; 20.3)
Total physical activity^c^, *hours/day*	3.1 (2.8; 3.4)***	4.3 (4.0; 4.6)	3.6 (3.7; 4.2)
Total MVPA^c^, *hours/day*	0.5 (0.7; 1.4)	0.5 (0.7; 1.0)	0.5 (0.7; 1.0)
Total MVPA^c^, *METs/day*	123.4 (160.8; 285.2)	119.0 (151.6; 213.3)	123.4 (166.4; 223.3)
Screen-based sedentary^c^, *hours/day*	4.0 (3.9; 4.7)	4.0 (3.8; 4.4)	4.0 (3.9; 4.2)
Blood interleukin-6^c^, pg/mL	1.68 (0.94; 2.42)***	2.83 (1.26; 3.49)	2.48 (1.96; 3.00)

*FA* fatty acids, *DXA* dual-energy χ-ray absorptiometry, *ASM* appendicular skeletal muscle mass, *MVPA* moderate to vigorous physical activity.

^a^Data only assessed in female participants.

^b^Values expressed as number of participants and percentage in parentheses.

^c^Values expressed as median and 95% CIs and statistical analysis based on log-*transformed data.*

Significant different from females at *P < 0.05; **P < 0.01 and ***P < 0.001.

### Assessment of sarcopenia risk

Based on the several diagnostic criteria methods used to assess sarcopenia risk, we decided to use that of the Asian Working Group for Sarcopenia 2019^1^ for the chosen population. The reason for choosing the 2019 AWGS’s diagnosis criteria is simply because it reflects the characteristics of the present population of Asia origin and is comparable to the method used to assess sarcopenia risk. The prevalence of sarcopenia and the proportion of components of participants are given in Table 3. About 13.9% were at risk of low muscle strength, as defined by a handgrip muscle strength of less than 28 kg and 18 kg for males and females, respectively. The prevalence of low muscle mass, as assessed by a height-adjusted appendicular muscle mass of <7.0 kg/m^2^ and <5.4 kg/m^2^ for males and females, respectively, was 26.1%, in which females had a significantly higher risk of low muscle mass than male participants (*P* < 0.01). The prevalence of sarcopenia was 12.6%, based on both indicators of muscle mass and handgrip muscle strength, as recommended by the newly revised diagnostic criteria for Asian populations^1^, with no significant difference found between males and females.

**Table 3:** Prevalence of sarcopenia based on the revised diagnostic criteria recommended by the Asian Working Group for Sarcopenia (AWGS 2019).

AWGS	Prevalence, *n* (%)		
	Male (n = 70)	Female (n = 160)	Total (N = 230)
Low muscle strength	11 (15.7)	21 (13.1)	32 (13.9)
Low muscle mass (ASM/height^2^)^a^	10 (14.3)	50 (31.3)**	60 (26.1)
Sarcopenia	9 (12.9)	20 (12.5)	29 (12.6)

Significant difference from females at **P < 0.01.

### Associations between sarcopenia and age, dietary and lifestyle factors

Further analysis was carried-out to assess associations between sarcopenia risk and age, dietary, and lifestyle factors (Table 4). It found that age was significantly higher among those participants at risk of sarcopenia (*P* < 0.01). Participants without sarcopenia had a significantly higher body weight (*P* < 0.0001), BMI (*P* < 0.0001), waist circumference (*P* < 0.01), DXA-derived lean body mass (*P* < 0.01), and total body fat (*P* < 0.01) as compared with those with sarcopenia. In terms of dietary and lifestyle behavioural practices, no significant differences were found between genders.

**Table 4:** Relationships between sarcopenia risk and all known exposure risk factors in categorical variables of male and female participants (n = 230).

	Sarcopenia risk		*P* values
	No (n = 201)	At Risk (n = 29)	
	Mean ± SD		
Age, *years*	57.6 ± 6.3**	62.0 ± 8.5	0.001
Age of menopause^a^, *years*	31.3 ± 24.4	35.0 ± 24.4	NS
Years since menopause^a^, *years*	5.1 ± 6.3	6.5 ± 7.3	NS
*Body composition variables*			
Body weight, *kg*	60.5 ± 11.7***	51.6 ± 6.8	*P* < 0.0001
Height, *m*	1.6 ± 0.1	1.6 ± 0.1	NS
Body mass index, *kg/m*^*2*^	23.8 ± 3.8***	20.9 ± 2.3	0.001
Waist circumference, *cm*	83.5 ± 10.1**	78.2 ± 7.9	0.007
DXA-lean body mass, *kg*	38.8 ± 8.6**	33.1 ± 5.5	0.005
DXA-body fat mass, *kg*	20.7 ± 6.1**	17.4 ± 4.3	0.005
*Dietary variables*^*b*^			
Energy, *Kcal/d*	1850.6 (1823.1; 1979.8)	1710.0 (1655.9; 1986.3)	NS
Protein, *g/d*	66.6 (66.2; 73.7)	61.1 (58.4; 72.2)	NS
Fat, *g/d*	50.6 (49.6; 57.2)	43.6 (39.6; 54.3)	NS
Omega 3 FA, *g*	1.5 (1.5; 1.9)	1.1 (1.0; 1.7)	NS
Omega 6 FA, *g*	3.0 (3.1; 3.6)	2.5 (2.3; 3.4)	NS
Vitamin D, *μg*	3.8 (4.1; 4.9)	3.0 (3.1; 5.5)	NS
Calcium, *mg*	518.8 (513.3; 584.9)	434.8 (396.8; 571.3)	NS
*Lifestyle factors*^*b*^			
Total physical activity, *hours/day*	3.7 (3.6; 4.1)	3.5 (3.5; 5.3)	NS
Total MVPA, *hours/day*	0.5 (0.8; 1.1)	0.4 (0.3; 1.0)	NS
Total MVPA, *METs/day*	128.6 (170.0; 232.3)	111.4 (83.5; 218.2)	NS
Screen-based sedentary, *hours/day*	4.0 (3.9; 4.4)	3.5 (3.3; 4.6)	NS

*NS* not significant.

^a^Data only assessed in female participants.

^b^Values expressed as median and 95% CIs and statistical analysis based on log-*transformed data.*

Significant difference from participants with a risk of sarcopenia at *P < 0.05; **P < 0.01 and ***P < 0.001.

### Determinant factors of age, pro-inflammatory cytokine level and body composition on sarcopenia

Since several factors were significantly associated with sarcopenia risk in these populations, we stratified the risk by age, body composition indicators of BMI and percentage of body fat and blood IL-6 concentration in both unadjusted simple and multivariate adjusted logistics regression models (Table 5). The risk of sarcopenia increased with advancing of age such that participants older than 70 years had an increased risk at 8.7 times [Adjusted OR: 8.7; 95% CI 2.21–35.5, *P*_*for trend*_ < 0.01], as compared to their younger counterparts, after adjustments for potential confounding factors of body composition profile and blood IL-6 status. In addition, participants with a higher blood IL-6 status had an increased likelihood of having sarcopenia at 2.3 times compared to than those of a low IL-6 status [Adjusted OR: 2.3; 95% CI 0.80–6.40; P_*for trend*_ < 0.05], after adjustments for age, and other body composition factors. On the contrary, there was no significant influence of either gender or body composition indicator assessed such as body mass index and percentage of body fat (*P*_*for trend*_ > 0.05) on the risk of sarcopenia after taking into account other factors such as age and blood IL-6 concentration. Moreover, no significant trends of interaction were observed between age and gender on the risk of sarcopenia among these participants.

**Table 5:** Relationships between sarcopenia risk and all known exposure risk factors of male and female participants (n = 230).

	Unadjusted Odds Ratio (95%CI)	*P* values	Adjusted Odds Ratio (95%CI)	*P* values
Prevalence of sarcopenia	12.6%			
Age, *years*				
< 60 years	1.00 (Referent)		1.00 (Referent)	
60–70 years	0.95 (0.37–2.44)	0.912	1.15 (0.39–3.36)	0.804
> 70 years	5.97 (1.98–18.1)	0.002	8.86 (2.21–35.5)	0.002
Gender				
Female	1.00 (Referent)		1.00 (Referent)	
Male	1.03 (0.45–2.40)	0.940	0.75 (0.25–2.21)	0.595
Body mass index				
Underweight	1.75 (0.51–5.93)	0.372	3.17 (0.75–13.36)	0.117
Normal	1.0 (Referent)		1.00 (Referent)	
Overweight and obese	0	0.997	0	0.996
DXA-Percentage Body fat, *%BF*				
Low	1.00 (Referent)		1.00 (Referent)	
High	0.93 (0.42–2.09)	0.866	2.26 (0.80–6.38)	0.124
Blood interleukin-6 concentrations, *pg/mL*				
Low	1.0 (Referent)		1.0 (Referent)	
High	2.38 (1.04–5.49)	0.041	2.59 (1.04–6.46)	0.042

^1^Adjusting for age (0 =  < 60 years, 1 = 60-70 years and 3 =  > 70 years), gender (0 = female; 1 = male), body mass index (0 = Underweight < 18.5 kg/m^2^; 1 = Normal (18.5–24.9 kg/m^2^) and 2 = overweight and obese (≥ 25.0 kg/ m^2^), percentage body fat (0 = low and 1 = high) and blood interleukin-6 concentrations (0 = low based on values lower than gender-specific median and 1 = high levels based on the values above the gender-specific median).

## Discussion

The main findings of this study showed that the prevalence of sarcopenia among apparently healthy middle-aged and older adults of Chinese-origin was 12.6%, consistent with numerous population-based epidemiological studies of older populations such as multi-ethnic adults aged 50 years and older at 19.3% in western China^6^, older adults aged 65 years and above at 12.7% of males, 13.3% of women in Taiwan^20^, community-dwelling pre- and postmenopausal women at 11.7% in the United States^28^ and women aged 40 years and older at 16.1%^29^. The prevalence of sarcopenia reported in Japanese women was slightly higher, potentially because their populations were recruited from orthopedic clinics already showing the presence of some form of skeletal disorder. In a recent study of multi-ethnic community-dwelling Chinese adults aged 50 years and older^6^, sarcopenia was assessed using the criterion method of the AWGS as used in the present study, showing at the prevalence of sarcopenia at 19.3%, with muscle mass assessed using a bioelectrical impedance analysis (BIA) device, slightly higher than that of the present study population. Although the similar diagnostic criterion of AWGS was used in both studies, the discrepancy in prevalence of sarcopenia between studies may be attributed to population characteristics such as ethnicity, genetic background and nutritional status and living environment. A possible explanation for the variation in prevalence rates of sarcopenia between both studies of Chinese populations could be attributed to differences of mean age and the diagnostic criterion methods used to assess muscle mass. It has been reported that muscle mass assessed by the BIA method is highly dependent on the degree of muscle mass^30,31^ since, the BIA method tends to overestimate it among participants with a high muscle mass and underestimate it in those with a low muscle mass. This could possibly be attributed to a higher prevalence of sarcopenia among the older populations in China. Furthermore, a similar pattern of sarcopenia risk was found between males and females in the present study, even though the male participants were older than the females.

We further examined the factors associated with sarcopenia risk, taking into account all potential known confounding factors such as age, and a body composition profile such as BMI and body fatness indicators. The results of the present study as well as some previous ones have reported that a high BMI and/or body fat may be significantly associated with greater muscle mass and strength^8,32,33^ as well as higher inflammation levels^12^. Therefore, a BMI and body fat percentage were included as important confounders to examine associations between all factors and sarcopenia risk among the participants. In the multivariate analysis, participants at an older age showed higher concentrations of the blood pro-inflammatory marker of IL-6 and a significantly increased risk of sarcopenia, after taking into consideration other potential factors such as gender and adiposity levels. These findings suggest that age and blood IL-6 concentrations emerged as significant independent determinants for the risk of sarcopenia among the older participants. Interestingly, only participants aged 70 years and above had an increased risk nine-fold risk of developing sarcopenia than their younger counterparts, after taking into account other potential confounding factors such as gender, and adiposity and blood IL-6 status. This finding is consistent with numerous population-based epidemiological studies conducted among other populations^9,12,34^, even though different diagnostic criterions were used to assess sarcopenia risk. Although it is generally agreed that the prevalence of sarcopenia increases with advancing age, it could be due to a rapidly accelerating loss of muscle mass and quality associated with aging since loss of muscle mass and strength are considered the risk factors of sarcopenia^1,2^. It is well established that the aging process leads to the deterioration of muscle mass and quality that may cause adverse effects in daily life such as reduced muscle strength and power and problems with mobility, consequently leading to an overall decline in metabolic function^35,36^. Moreover, it is reported that the onset of age-related loss of muscle mass can occur as early as 30 years, followed by a gradual decrease by 1 to 2% after the age of 50 years and approximately 50% by 80^37,38^.

An increasing body of evidence suggests that increased blood pro-inflammatory cytokines concentrations in the body play a major role in the pathogenesis of major chronic diseases, including sarcopenia^12,39^, through the activation of inflammatory signal pathways. In addition, several pro-inflammatory cytokines such as the IL-6 and tumor necrosis factor alpha (TNF-α) are shown to have an important influence on the modulation of the inflammation signaling pathway in the age-related loss of skeletal muscle^11^. In the present study, increased pro-inflammatory cytokine levels, as assessed by blood IL-6 concentrations, is significantly associated with a higher risk of sarcopenia even after further adjustments for age, gender and adiposity levels, suggesting that blood IL-6 concentrations might contribute to sarcopenia risk beyond its effects on age and body weight. The findings of the present study accord with previous studies^9,12,40^. The results of these and our present study support a direct relationship between cytokine levels and muscle mass during ageing, suggesting a potential role for chronic low-grade inflammation in the development of sarcopenia in healthy older adults. Complex interactions exit between inflammation and sarcopenia, and it is influenced by many mediating factors such as age, body composition and modifiable lifestyle environmental factors^41,42^. Further studies with a large sample size are needed to further elucidate the magnitude of changes in pro-inflammatory cytokine concentrations concerning the loss of muscle mass and strength associated with aging and whether they can be used as useful and practical diagnostic biomarker to assess the ageing-related atrophy of skeletal muscle as well as the screening and diagnosis of sarcopenia in near future.

Although a significant positive association between the pro-inflammatory cytokine marker and sarcopenia risk, whether such elevated pro-inflammatory cytokines on the causal pathway may be attributed to the development of muscular disability or a marker of underlying disease in the muscle loss associated with sarcopenia is still unclear^11,12,39^. Moreover, the precise roles of these pro-inflammatory cytokines in aging skeletal muscle have yet to be fully understood. Several mechanisms of action have been postulated to explain the positive relationship between the pro-inflammatory cytokines and sarcopenia risk during aging. Pro-inflammatory cytokines may be a crucial factor in the catabolic effect on muscle mass and muscle strength during aging, as indicated by a significant decline in functional disability and performance^2,43^. It clearly shows that an interplay exists between the loss of muscle mass and elevated systemic inflammation. In fact, sarcopenia is a complex process manifested by a subclinical state of inflammation driven by pro-inflammatory cytokines and oxidative stress that could increase the infiltration of immune cells into injured muscles. This inflammation subsequently aggravates muscle loss and fat accumulation in the aging skeletal muscle cell and consequently decreases muscle strength and function^11,12^.

Apart from the impact of age and blood IL-6 concentration on sarcopenia risk, modifiable factors such as dietary nutrient intake and lifestyle physical activity failed to reveal significant associated factors impacting the risk of sarcopenia in the present participants, although some significant associations were found between some nutrients of interests and the indices used for the muscular mass and strength, as assessed by the DXA and handgrip muscle dynamometer devices (Supplementary information). Therefore, several other dietary and lifestyle analysis approaches are currently being undertaken in lieu of individual, and specific nutrient and physical activity profiles on muscle mass and strength that accompanies aging. A significant decline muscle quantity and quality during aging may have direct impact on the risk of sarcopenia among older populations.

### Limitations and strengths of the study

The present study had several limitations. First, the cross-sectional nature of the study means that the cause of any effect cannot be addressed. Hence, a longitudinal study with a large sample size would be important to establish any causal mechanisms of sarcopenia risk among older Chinese adults. Another possible limitation was the reliance on a self-assessment of habitual dietary intake and lifestyle physical activity levels. However, the assessment of both lifestyle factors was part of a questionnaire validated among the present Chinese populations in Kelantan. Moreover, each participant was assisted by trained personnel throughout the completion of physical activity and dietary intake to minimise assessment errors. Lastly, participants included in the present study were relatively healthy as we excluded those diagnosed with any form of diagnosed health condition such as hypertension, diabetes, any bone skeletal disorders, cardiovascular diseases and cancers. The present participants may be in better health than the entire older populations and this may have been biased compared with several other studies of mixed older populations of healthy and also with some form of health disorders. Hence, the findings of the present study are limited to apparently healthy older populations.

Despite limitations, the present study has a number of strengths. First, this study is one of the first to use the newly revised Asian Working Group for Sarcopenia diagnostic criteria and the more reliable and accurate assessment tool of DXA to assess muscle mass in the prevalence of sarcopenia in Malaysian populations. Second, in terms of design, the present study used more comprehensive data on body composition, menopausal status, muscle strength, dietary intake, lifestyle practices, and blood biomarkers assessments not previously evaluated in previous studies. The data obtained provided comprehensive insight into factors associated with sarcopenia risk among the older Chinese adults. Third, the use of DXA is considered the gold standard in assessing the quantity and composition of muscle; it can detect small changes over time compared to other diagnostic methods such as BIA^30,44^. Last, this study was conducted with a reasonably large sample size of apparently healthy community-dwelling middle-aged and older adults of Chinese origin living in community-based areas, where they are relatively physically active. The findings may now be used to formulate effective nutrition and lifestyle health promotion strategies for the prevention of rapid muscle loss to subsequently reduce the risk of sarcopenia during aging.

## Conclusions

Sarcopenia is considered to be an emerging public health problem and challenge worldwide, including Malaysia because it significantly affects the functional daily activities and quality of life of older populations. The findings of the present study show a reasonably high prevalence of sarcopenia risk at 12.6% among apparently healthy middle-aged and older Chinese adults. Furthermore, the present findings indicate that older adults aged 70 years and above with higher inflammation levels had a significantly greater sarcopenia risk. Hence, effective dietary and lifestyle intervention strategies should emphasize the reduction of inflammation associated with aging to prevent the rapid loss of muscle mass and strength that can lead to sarcopenia. Moreover, prospective studies with a large sample size will further elucidate the natural progression and predisposing modifiable dietary and lifestyle factors associated with the decline in muscle mass and strength that occur with aging. More targeted dietary and lifestyle interventions can be formulated to prevent or slow the decline of muscle mass and strength that consequently lead to sarcopenia among older adults.

## Supplementary Information

Supplementary Table S1.

## Data Availability

The dataset used and/or analysed for the present study is available from the corresponding author on reasonable request. All data underlying the findings of the study are included in this published article.
