# Supplementary material for: Assessments of sarcopenia and its associated factors in community-dwelling middle-aged and older Chinese adults in Kelantan, Malaysia
Source: Sci Rep. 2023 May 9;13:7498. doi: 10.1038/s41598-023-34668-w (PMC10170113; doi:10.1038/s41598-023-34668-w)
Supplement: Supplementary file 1 — Supplementary Table S1. [file 41598_2023_34668_MOESM1_ESM.pdf]

Supplementary table S1. Relationships between the height-adjusted DXA-derived appendicular skeletal muscle mass (Height-adjusted ASM) and handgrip muscle strength, and dietary factors (n=230)

	Height-adjusted ASM	Handgrip muscle strength
	r value	
Dietary variables[a]		
Energy, *Kcal/d*	0.173**	0.183**
Protein, *g/d*	0.085	0.116
Fat, *g/d*	0.076	0.069
MUFA, *g*	0.096	0.074
PUFA, *g*	0.043	0.053
Vitamin C, *mg/d*	-0.027	0.004
Vitamin D, *μg*	0.209**	0.132*
Calcium, *mg*	0.062	0.088
Magnesium, *mg*	-0.013	0.037
Zinc, *mg*	0.124	0.107
Selenium, μg	0.092	0.133*
Potassium, *mg*	0.058	0.099

Abbreviations: MUFA, monounsaturated fatty-acids; PUFA, polyunsaturated fatty-acids

[a]Statistical analysis based on log-*transformed data*

Significant correlation at *p<0.05 and **p<0.01
